# Context-dependent lysophosphatidic acid signalling in inflammation: evidence hierarchy, myeloid regulation and translational gaps

**DOI:** 10.1007/s00011-026-02337-z

**Published:** 2026-08-04

**Authors:** Wataru Nagata, Kunio Takada

**Affiliations:** Division of Environmental Medicine, National Defense Medical College, Tokorozawa, Saitama, 359-8513 Japan

**Keywords:** Lysophosphatidic acid, Autotaxin, LPA receptor, Macrophage, Microglia, Immunometabolism

## Abstract

**Background:**

Lysophosphatidic acid (LPA) has been extensively reviewed in receptor pharmacology, fibrosis, cancer, vascular biology and neural injury. Its role in inflammation remains difficult to interpret because well-replicated pathogenic or pro-remodelling actions coexist with selected macrophage-regulatory effects reported under defined experimental conditions.

**Findings:**

This structured narrative review provides an evidence-weighted framework for interpreting these divergent findings. The strongest evidence supports LPA as an injury- and remodelling-associated signal in vascular–stromal programmes, pain-associated settings and tumour immune escape. By contrast, LPA-mediated attenuation of selected LPS/TLR4-driven macrophage responses is biologically plausible but incompletely replicated and receptor-divergent. The more specific LPAR1-dependent model of M2-like and metabolic macrophage reprogramming remains less independently validated, especially in primary human myeloid cells and physiologically plausible concentration ranges.

**Conclusions:**

LPA should not be classified as simply pro-inflammatory or anti-inflammatory. Anti-inflammatory LPA models should be regarded as experimentally plausible but translationally unproven until validated using defined LPA species, receptor-resolved perturbation and concentration-resolved human myeloid-cell systems.

**Supplementary Information:**

The online version contains supplementary material available at 10.1007/s00011-026-02337-z.

## Introduction

Inflammation proceeds through initiation, amplification, containment, repair and, in some settings, chronic maladaptive remodelling. Lipid mediators regulate several of these phases by controlling vascular permeability, leukocyte recruitment, stromal activation, cytokine production and tissue repair. Lysophosphatidic acid (LPA) is one such mediator. It is generated predominantly through autotaxin (ATX)-mediated conversion of lysophosphatidylcholine to LPA, although platelet activation, phospholipase activity and tissue injury can contribute additional sources [1–5]. LPA signals through six established G protein-coupled receptors, LPAR1-LPAR6, which activate Rho/ROCK, PI3K-Akt, MAPK/ERK, calcium-dependent and cytoskeletal pathways [6–8].

The literature on LPA in inflammation is marked by an apparent paradox. A substantial body of evidence supports pathogenic roles for LPA across several distinct inflammatory contexts: leukocyte recruitment and activation [9–13], vascular leak and fibrotic remodelling [14, 15], tumour immune escape [16, 17], neuropathic pain and CNS injury-associated signalling [18–22], and mast-cell activation through LPAR5 [23]. Clinical experience also underscores the difficulty of translating broad ATX–LPA pathway inhibition into antifibrotic benefit [24], while tumour immune-evasion framing [25] and human synovial-fluid and synovial-tissue studies [26–29] further illustrate the clinical relevance of pathogenic or remodelling-associated LPA signalling. Human cerebrospinal fluid (CSF) studies further support an injury-associated LPA signal after traumatic brain injury [30, 31].

By contrast, selected experimental studies suggest that LPA can attenuate LPS/TLR4-driven macrophage activation, increase IL-10, suppress TNF-alpha and promote regulatory or M2-like myeloid features under defined conditions [32–35]. This second body of evidence is biologically important but less extensively replicated. LPA-mediated attenuation of macrophage inflammatory responses is independently supported at the level of the phenomenon [32, 33], but the receptor mechanisms differ across studies. The more specific LPAR1-linked macrophage-regulatory model is supported by a smaller and less independently replicated body of evidence and should be considered hypothesis-generating until validated in primary human myeloid cells and disease-relevant settings [35–37].

Several recent reviews have addressed LPA biology broadly, including receptor pharmacology, cancer, fibrosis, vascular biology, neural injury and inflammatory disease [1, 2, 6–8, 38, 39]. Rather than cataloguing LPA actions comprehensively, this review asks why divergent inflammatory effects arise and how established evidence should be separated from preliminary mechanistic models. Four issues are central to this approach: the asymmetry between strong pathogenic LPA evidence and more limited anti-inflammatory myeloid evidence; the translational gap between micromolar in vitro LPA exposure and nanomolar to low-micromolar human biofluid concentrations; the need to distinguish general LPA-mediated attenuation of TLR4 responses from receptor-specific LPAR1 models; and the requirement for falsifiable predictions that can test rather than merely describe context-dependent LPA signalling.

**Fig. 1:**
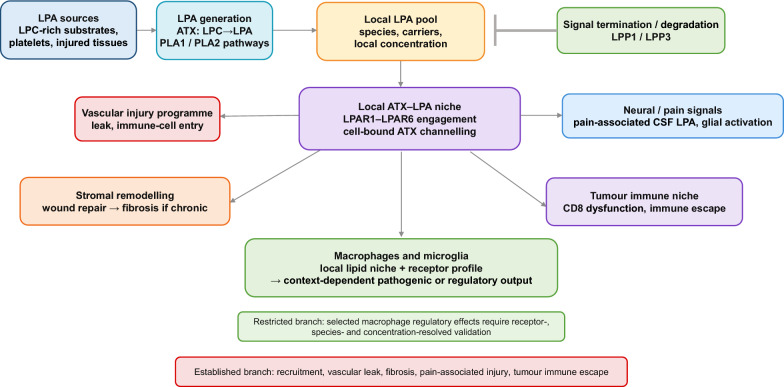
Vascular–stromal and myeloid interfaces in LPA-driven inflammation. LPC-rich substrates, platelets and injured tissues provide LPA-related inputs. LPA is generated through ATX-dependent LPC-to-LPA conversion and PLA1/PLA2-associated phospholipase pathways, and the local LPA pool is shaped by LPA species, carriers and local concentration. Signal termination / degradation is mediated by lipid phosphate phosphatases, including LPP1 and LPP3. Secreted or cell-bound ATX can support local LPA channelling to nearby receptors. Together, these processes define a local ATX–LPA niche in which LPAR1–LPAR6 engagement drives context-dependent vascular, stromal, neural and immune outputs. Established branches include recruitment, vascular leak, fibrosis, pain-associated injury and tumour immune escape. Selected macrophage and microglial regulatory outputs are shown as a restricted branch requiring receptor-, species- and concentration-resolved validation

Macrophages and microglia do not respond to LPA in isolation. Their responses are shaped by vascular permeability, stromal remodelling, platelet activation, neural injury and tumour-derived lipid signals. For this reason, vascular–stromal and human biofluid data are discussed as upstream determinants of myeloid LPA responses rather than as separate topics (Fig. 1).

The central argument of this review is that the inflammatory output of LPA is determined not by LPA itself, but by the combination of tissue niche, receptor subtype, ligand species, local concentration, disease phase and myeloid-cell state. This review therefore does not aim to reclassify LPA as an anti-inflammatory lipid mediator. Instead, it proposes an evidence hierarchy that separates: first, independently replicated pathogenic LPA functions in vascular–stromal injury programmes; second, independently supported but receptor-divergent attenuation of TLR4-driven macrophage responses; third, author-group-limited LPAR1-dependent macrophage-regulatory models; and fourth, hypothesis-only translational claims that remain untested in human disease.

## Literature search and evidence-classification approach

This is a structured narrative review rather than a formal systematic review or meta-analysis. PubMed-indexed English-language studies were searched from database inception to May 2026. Search terms included ‘lysophosphatidic acid’, ‘LPA’, ‘autotaxin’, ‘lipid phosphate phosphatase’, ‘LPAR1’, ‘LPAR2’, ‘LPAR5’, ‘LPAR6’, ‘macrophage’, ‘microglia’, ‘TLR4’, ‘LPS’, ‘‘IL-10’, ‘TNF-alpha’, ‘neuroinflammation’, ‘cerebrospinal fluid’, ‘synovial fluid’, ‘rheumatoid arthritis’, ‘lupus’, ‘fibrosis’, ‘vascular permeability’, ‘tumour immunity’, ‘cancer immunity’, ‘immunometabolism’, ‘cyclic phosphatidic acid’ and ‘2-carba cyclic phosphatidic acid’. Additional targeted searches were performed for human disease-tissue or biofluid studies using combinations of ‘lysophosphatidic acid’ or ‘autotaxin’ with ‘rheumatoid arthritis synovial fluid’, ‘lupus nephritis kidney’, ‘multiple sclerosis cerebrospinal fluid’, ‘amyotrophic lateral sclerosis cerebrospinal fluid’, ‘Parkinson disease cerebrospinal fluid’ and ‘inflammatory bowel disease intestinal tissue’.

Studies were prioritised when they directly addressed inflammatory function, receptor subtype, LPA species, experimental concentration, human biospecimen relevance, independent replication or disease phase. Non-English reports, abstracts without accessible full text and studies without clear inflammatory or mechanistic relevance were not emphasised. Because the maturity of the evidence varied substantially across disease contexts, studies were categorised as independently replicated, independently supported but receptor-divergent, author-group limited or hypothesis-only. This classification was used to avoid conflating a general phenomenon, such as LPA-mediated attenuation of LPS/TLR4 responses, with a narrower receptor-specific model, such as LPAR1-dependent M2-like macrophage reprogramming.

## LPA production, degradation and inflammatory niches

LPA consists of a glycerol backbone, a phosphate group and one fatty acyl chain. Individual LPA species differ in acyl-chain length, saturation and linkage, and these differences can influence receptor potency, receptor preference, tissue distribution and biological output [1, 2, 39]. Common experimental species include 16:0, 18:0, 18:1 and 20:4 LPA. The frequent use of 18:1 LPA in vitro provides experimental standardisation but does not necessarily reflect the full species composition present in inflamed human tissues.

The major extracellular pathway for LPA generation is mediated by ATX, which converts lysophosphatidylcholine to LPA [3–5]. In addition to the ATX pathway, phospholipase-dependent routes can contribute to LPA generation. Phospholipase A1 and phospholipase A2 isoenzymes can generate LPA by deacylating phosphatidic acid, and phospholipase A2-mediated lysophosphatidylcholine production can also increase substrate availability for ATX [4, 40]. ATX can be produced by stromal cells, adipocytes, endothelial cells, fibroblasts and inflammatory tissue compartments. During tissue injury, platelet activation, phospholipase activity, cell death and extracellular matrix remodelling can further alter local LPA production. LPA is degraded by lipid phosphate phosphatases, including LPP1 and LPP3, which help determine local signalling duration and receptor exposure [41–45]. Although LPP2 is also a mammalian LPP family member [42], its specific contribution to inflammation-associated or macrophage-centred LPA signal termination is less clearly established, and this review therefore focuses on LPP1 and LPP3.

Inflammatory cytokines and local lipid mediators can further amplify this system by modulating ATX transcription and secretion, thereby linking tissue inflammation to enhanced extracellular LPA production [29, 46–48]. In rheumatoid arthritis-related stromal systems, TNF-driven ATX expression and LPA-dependent fibroblast activation provide a disease-relevant example of this coupling [29]. LPA receptor signalling can in turn promote additional cytokine, chemokine and eicosanoid production in susceptible stromal, vascular or immune compartments, establishing an ATX-LPA-inflammatory feedforward cycle. This cycle should not be interpreted as uniformly pathological: during acute tissue injury, transient ATX-LPA signalling may support cell migration, vascular adaptation and wound repair, whereas persistent activation in chronic inflammatory or fibrotic niches may maintain maladaptive cytokine production, vascular leakage, stromal remodelling and tissue damage.

These metabolic pathways are important for myeloid-cell biology because macrophages and microglia respond to local lipid niches rather than to systemic LPA alone. In inflamed tissues, vascular leakage, platelet activation, stromal ATX production and impaired LPA degradation may increase local LPA exposure around infiltrating macrophages [3–5, 14, 41, 42]. Conversely, the same tissue compartment may contain albumin-bound LPA, multiple LPA species and receptor-modifying inflammatory cytokines. The relevant signalling variable is therefore not simply total circulating LPA, but the local combination of LPA species, free versus carrier-bound lipid, ATX activity, lipid phosphate phosphatase activity, receptor expression and cellular activation state.

A further implication is that LPA signalling is highly local. Secreted ATX can bind to cell-surface structures, including integrins, and thereby generate or channel LPA near adjacent receptors rather than acting only as a freely diffusible enzyme in bulk extracellular fluid [49, 50]. Consequently, ATX or LPA concentrations measured in plasma, sputum, CSF or other biofluids may indicate increased systemic or compartmental inflammation, but they do not necessarily report the intensity, receptor selectivity or cellular location of active ATX-LPA signalling within tissue niches.

This niche perspective also explains why vascular–stromal LPA biology is directly relevant to myeloid inflammation. LPA-driven endothelial permeability can increase immune-cell entry into tissue [14]. Fibroblast and stromal responses can alter chemokine gradients, extracellular-matrix stiffness and retention signals for macrophages [14, 15, 27–29]. Tumour-derived or stromal LPA can suppress cytotoxic lymphocyte function while shaping immune-cell polarisation [16, 17, 25]. Thus, vascular and stromal LPA programmes should be interpreted as upstream modifiers of the myeloid inflammatory environment rather than as unrelated LPA effects.

## LPA receptor biology relevant to inflammation

The six established LPA receptors provide a mechanistic basis for divergent inflammatory responses. LPAR1 is widely expressed and couples to pathways that regulate migration, cytoskeletal dynamics, survival and permeability [6–8]. In vascular and fibrotic contexts, LPAR1 promotes fibroblast recruitment, vascular leak and tissue remodelling [14, 15]. In selected macrophage models, however, LPAR1-associated signalling has been linked to cytokine suppression and M2-like features [35]. This contrast does not imply a universal anti-inflammatory role for LPAR1; rather, it illustrates that receptor output is cell-state dependent.

LPAR2 has been implicated in epithelial repair, barrier regulation and broader GPCR scaffolding biology, but direct evidence for LPA2-dependent non-canonical scaffolding mechanisms in macrophage LPA signalling is lacking. Therefore, LPAR2-related non-canonical pathways are not used here as explanatory anchors and should be viewed only as candidate modules for future testing.

LPAR3 and LPAR4 are included in Table 1 for completeness, but current evidence linking these receptors to myeloid-specific LPA regulation is comparatively limited. In this review, they are therefore treated as potential contextual modifiers rather than central drivers of the macrophage-regulatory model.

**Table 1:** LPA receptor subtypes relevant to inflammation

Receptor	Major signalling emphasis	Inflammation-relevant contexts	Interpretation in this review
LPAR1	Gi/o, Gq, G12/13; Rho/ROCK, PI3K-Akt, MAPK	Fibroblasts, endothelial cells, macrophages, neural and vascular compartments	Strong evidence for fibrosis and vascular leak; LPAR1-dependent macrophage regulation remains author-group limited
LPAR2	Gi/o, Gq, G12/13; epithelial and barrier signalling	Epithelial and stromal compartments; possible immune signalling	Not established in macrophage LPA regulation; non-canonical scaffolding and metabolic pathways remain speculative
LPAR3	Gi/o and Gq; calcium and migration-related signalling	Stromal cells and leukocyte recruitment contexts	Included for completeness; not central to macrophage-regulatory evidence
LPAR4	G12/13 and Gs-related signalling; cytoskeletal regulation	Vascular and stromal contexts; less defined in myeloid inflammation	Potential modifier of vascular–stromal niches; limited direct myeloid evidence
LPAR5	G12/13, Gq and Gi-related signalling; MAPK/PKD pathways	Mast cells, microglia, CD8 T cells, tumour immunity, macrophage IL-10 responses	Relevant to pro-inflammatory microglia and tumour immune escape; also implicated in receptor-divergent macrophage IL-10 induction
LPAR6	G12/13-related signalling; context-dependent macrophage responses	Macrophage cytokine regulation in selected systems	Implicated with LPAR5 in independent macrophage IL-10/TNF-alpha studies; not equivalent to the LPAR1 model

## Evidence hierarchy and reproducibility

**Fig. 2:**
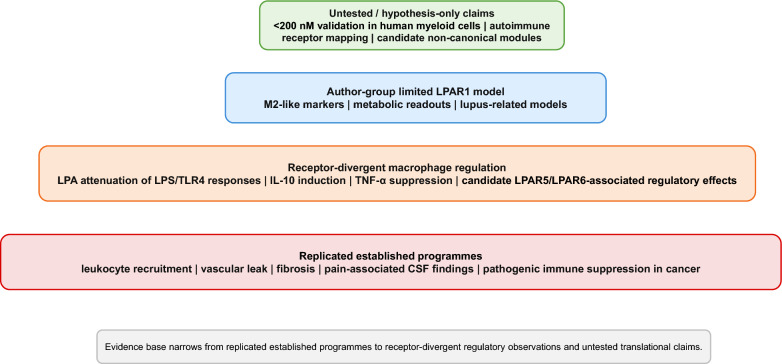
Evidence hierarchy for context-dependent LPA signalling. The evidence pyramid separates four levels of support. The base contains replicated established programmes, including leukocyte recruitment, vascular leak, fibrosis, pain-associated CSF findings and pathogenic immune suppression in cancer. The middle layer contains receptor-divergent macrophage regulation, including LPA attenuation of LPS/TLR4 responses, IL-10 induction, TNF-α suppression and candidate LPAR5/LPAR6-associated regulatory effects. The upper layer contains author-group-limited LPAR1-dependent macrophage-regulatory models, including M2-like and metabolic reprogramming in selected autoimmune-related experimental systems. The apex contains untested or hypothesis-only claims, including validation at < 200 nM LPA in human myeloid cells, human autoimmune receptor mapping and candidate non-canonical signalling modules. Overall, the evidence base narrows from replicated established programmes to receptor-divergent macrophage-regulatory observations and untested translational claims

Because LPA biology spans strong disease-promoting evidence, receptor-divergent regulatory evidence and author-group-limited mechanistic models, the field requires an explicit weight-of-evidence hierarchy. After outlining LPA production and receptor biology, the evidence hierarchy is summarised to make the evidentiary weight of each claim explicit before disease-specific and translational interpretations are considered. Table 2 separates independently replicated disease-promoting actions from limited anti-inflammatory macrophage phenomena and untested translational claims; the same hierarchy is summarised visually in Fig. 2.

**Table 2:** Evidence hierarchy and weight of evidence for LPA actions in inflammation

Evidence domain	Direction	Weight of evidence	Interpretation
Leukocyte recruitment, vascular leak, fibrosis, pain-associated CSF findings and tumour immune escape	Pathogenic remodelling or pathogenic immune suppression	Strong independent evidence	Established injury-associated or immune-evasion programmes; not evidence of inflammation resolution
General LPA attenuation of LPS/TLR4 macrophage responses	Regulatory under selected conditions	Independent but limited; receptor-divergent	Supported as a phenomenon, but receptor mechanisms differ across studies and often involve pharmacological concentrations
LPAR1-dependent M2-like and metabolic macrophage regulation	Regulatory in selected experimental systems	Author-group limited; preliminary	Requires independent validation in primary cells, disease models and physiologically plausible concentration ranges
Murine lupus-related LPA or LPA-analogue studies	Regulatory in selected disease models	Author-group limited disease-model evidence	Hypothesis-generating autoimmune data; human translation is not established
Human primary macrophage regulation at < 200 nM LPA	Unknown	Untested	Major translational gap; no receptor-resolved human primary macrophage dose–response study has demonstrated TNF-alpha suppression below 200 nM
Non-canonical scaffolding in LPA-driven macrophage regulation	Unknown	Hypothesis-only	Should not be depicted as an established LPA macrophage mechanism

This structure is not intended to weaken the biological importance of macrophage-regulatory LPA effects, but to prevent overgeneralisation. Treating all positive LPA findings as equivalent would obscure a central translational problem: the strongest human data currently constrain, rather than directly support, anti-inflammatory LPA models.

## Established pro-inflammatory and pro-remodelling actions of LPA

The strongest evidence in the LPA inflammation literature supports pathogenic or pro-remodelling functions. LPA promotes eosinophil and neutrophil chemotaxis, Ca2 + -mobilization, actin reorganization, oxidative responses and CD11b up-regulation [9–13]. Although several of these studies are older, they are retained here as landmark mechanistic demonstrations of direct leukocyte responses to LPA and are interpreted together with more recent reviews and disease-oriented studies of the ATX-LPA axis [1, 38, 51]. These acute leukocyte effects provide a clear mechanism by which LPA can amplify early inflammatory recruitment.

LPA also regulates vascular permeability and fibrotic remodelling. In lung injury and pulmonary fibrosis models, LPAR1 links tissue injury to fibroblast recruitment and vascular leak [14]. LPAR1 antagonism attenuates fibrosis in experimental systems, supporting a pathogenic role for sustained LPA signalling in maladaptive repair [15]. These findings are relevant to myeloid inflammation because vascular leak and stromal remodelling alter macrophage entry, retention and activation within injured tissue.

In cancer, LPA can promote tumour progression and immune escape. LPA signalling can impair CD8 T-cell activation and antitumour immunity, and tumour or stromal LPA may create an immunosuppressive microenvironment [16–18, 25]. Although this is not a macrophage-specific mechanism, it demonstrates how LPA modifies immune niches that influence myeloid and lymphoid function.

In the nervous system, LPA has been associated with neuropathic pain, glial activation and injury-related signalling. Experimental studies show that LPA can promote pro-inflammatory microglial phenotypes through LPAR5 and MAPK-related pathways [19, 20]. These data contrast with selected autoimmune mouse studies in which LPA-related interventions suppress microglial activation [36, 37, 52, 53]. The discrepancy is not a minor inconsistency; it is a central feature of LPA biology. Acute injury, pain and tissue disruption appear to favour pathogenic LPA actions, whereas selected chronic immune models may reveal different myeloid regulatory responses under defined treatment conditions.

## Human biofluid data as translational constraints

Human biofluid data should be interpreted primarily as translational constraints rather than as direct support for anti-inflammatory LPA models. Available CSF and synovial-fluid studies generally associate LPA or LPA-related lipid species with injury, pain, tissue remodelling or disease activity. In traumatic brain injury, CSF LPA rises during the early post-injury period, consistent with an injury-associated lipid signal [30, 31]. In lumbar spinal stenosis and neuropathic pain-related contexts, CSF LPA or related lysophospholipid species have been associated with symptom severity [18]. These findings do not establish LPA as the sole mediator of CNS pathology, but they argue against a simple neuroprotective or inflammation-resolving interpretation of LPA in human CNS disease. Representative human biofluid contexts are also summarised in Supplementary Table S1.

Similarly, synovial-fluid and synovial-tissue data in rheumatoid arthritis support a role for the ATX–LPA axis in fibroblast-like synoviocyte migration, tissue remodelling and inflammatory-niche formation [27–29]. These human data are more consistent with pathogenic or remodelling-associated LPA activity than with inflammation resolution. To date, no human disease study has demonstrated that LPA promotes inflammation resolution or M2 macrophage polarisation in vivo. The absence of human resolution-phase evidence remains a central limitation of the field, not a minor technical gap.

A targeted narrative search for human disease-tissue LPA measurements identified relevant rheumatoid arthritis synovial-fluid and synovial-fibroblast studies, and literature discussing ATX–LPA biology in systemic lupus erythematosus [27–29, 54]. However, receptor-resolved and species-resolved LPA data remain sparse for human lupus nephritis kidney tissue, multiple sclerosis CSF, neurodegenerative-disease CSF and inflammatory bowel disease tissue. Where such data are absent or incomplete, this review treats the absence as a translational gap rather than inferring support from animal models. Kidney and vascular-injury studies provide additional organ-specific context for LPA biology, but they do not provide receptor-resolved validation of the macrophage-regulatory model in human lupus nephritis [55–57].

Autoimmune diseases may nevertheless differ from acute injury states in ways that are biologically relevant to LPA signalling. Chronic exposure to type I interferons, TNF-alpha, immune complexes, complement activation, barrier dysfunction and repeated tissue repair may alter ATX expression, LPP activity, LPA species composition and LPAR expression in resident or infiltrating myeloid cells [58–64]. These mechanisms could theoretically produce LPA responses that differ from those observed in acute injury or pain-associated biofluids. However, this remains a hypothesis requiring direct human validation rather than evidence for clinical protection.

## Anti-inflammatory macrophage effects: hierarchical interpretation

Evidence that LPA can attenuate macrophage inflammatory responses should be interpreted in layers. At the broadest level, LPA-mediated suppression of LPS/TLR4-driven inflammatory responses has independent support. Fan and colleagues reported that LPA suppressed endotoxin-induced pro-inflammatory responses in vivo and in peritoneal macrophages, with evidence implicating Gi-dependent signalling, PI3K, ERK1/2 and serine/threonine phosphatase pathways [33]. Ciesielska and colleagues further showed that LPA increased IL-10 production and reduced TNF-alpha synthesis in LPS-stimulated macrophages, with evidence suggesting involvement of LPAR5 and LPAR6 [32]. Mirzoyan and colleagues reported protective effects of LPA in endotoxin-induced acute kidney injury [34]. Together, these non-author-group studies support the general concept that LPA can limit selected TLR4-driven macrophage inflammatory responses under defined experimental conditions. LPA has also been reported to promote monocyte-to-macrophage conversion in both mice and humans, further illustrating that LPA can influence macrophage lineage and activation context [65].

However, these independent studies do not directly validate the specific LPAR1-dependent macrophage-regulatory model. In our recent RAW264.7 macrophage study, the LPAR1 antagonist AM095 reversed LPA-mediated TNF-alpha suppression, suggesting involvement of LPAR1 in that experimental system [35]. LPA exposure was also associated with reduced lactate, increased ATP and M2-like marker changes [35]. These data are hypothesis-generating rather than definitive evidence of an LPAR1-driven immunometabolic programme, because they have not yet been independently reproduced in primary human macrophages or tested across physiological LPA concentrations.

The most defensible hierarchy is therefore as follows: general LPA attenuation of LPS/TLR4 macrophage responses is independently supported; receptor-specific mechanisms are divergent across studies; LPAR1-dependent M2-like and metabolic reprogramming remains author-group limited; and relevance to primary human macrophages at physiologically plausible concentrations remains untested. The hierarchy helps prevent two opposing errors: dismissing all regulatory LPA effects as self-citation, and overgeneralising a receptor-specific author-group model to human inflammatory disease.

Several reviewer-relevant limitations should be stated explicitly. First, we do not currently have dose–response data demonstrating macrophage TNF-alpha suppression by LPA at concentrations below 200 nM. Second, independent support exists for LPA-induced IL-10 elevation and TNF-alpha suppression in LPS-stimulated macrophages, but this evidence implicates LPAR5 and LPAR6 rather than LPAR1. Third, LPA2-deficient macrophages have not been used to test LPA-mediated cytokine suppression in our experimental system, and non-canonical scaffolding mechanisms remain unvalidated in LPA-driven macrophage regulation. Fourth, receptor-resolved human validation studies combining LPA species measurement with LPAR1-LPAR6 expression in lupus nephritis kidney tissue or rheumatoid arthritis synovial fluid are not yet available. These gaps define the experimental boundaries within which the current macrophage-regulatory model should be interpreted.

## CNS inflammation and autoimmune models

Microglia provide a particularly important example of context-dependent LPA signalling. In experimental systems, LPA can induce motility and pro-inflammatory microglial activation through LPAR5 and MAPK-dependent pathways [19, 20]. These findings align with human CSF studies in which LPA or related lysophospholipids increase in injury- or pain-associated conditions [30, 31].

By contrast, selected murine autoimmune models suggest that LPA or LPA-related analogues can suppress microglial activation and improve behavioural phenotypes [36, 37, 52, 53]. These studies should be interpreted as model-specific rather than as evidence that LPA is broadly protective in human neuroinflammation. Chronic autoimmune neuroinflammation differs from acute traumatic injury or neuropathic pain in timing, lipid exposure, barrier status, receptor expression and immune-cell composition.

SLE provides a useful test case because it combines systemic immune activation, autoantibody production, vascular dysfunction, organ-specific inflammation and tissue-resident myeloid activation [58–64]. In lupus-related models, LPA should not be interpreted only as a macrophage-autonomous mediator. Endothelial cells, platelets, fibroblasts, lymphocytes and tissue stromal cells may generate or respond to LPA, thereby altering the local niche in which macrophages and microglia function. Murine lupus nephritis-like and neuropsychiatric lupus data from the authors’ programme suggest regulatory LPA-related effects [35–37], but these observations require independent disease-model replication and human validation.

## Concentration-dependent effects and translational plausibility

The concentration gap is not a technical detail but a central determinant of translational validity. Many in vitro studies use LPA at 1–10 µM, whereas human plasma, CSF or tissue-fluid measurements often fall in the nanomolar to low-micromolar range and are strongly influenced by compartment, platelet activation, sample processing and analytical method. Micromolar LPA exposure should not automatically be dismissed as artefactual or non-physiological, because LPA is actively degraded by cell-surface and extracellular LPP activity in vitro and is not continuously replenished during multi-hour incubations as it may be within an ATX-rich tissue niche in vivo [41–45]. If macrophage cytokine suppression is observed only at micromolar concentrations, the finding should be interpreted in relation to sustained exposure, high-local LPA exposure, LPP activity, ATX-mediated replenishment, carrier conditions and exposure duration rather than as a simple surrogate for circulating physiological concentration.

The appropriate concentration range also depends on the kinetic readout. Nanomolar or submicromolar LPA concentrations are particularly appropriate for rapid responses such as Ca2 + -mobilization or early ERK activation over seconds to minutes, when enzymatic degradation during the assay is limited. By contrast, multi-hour cytokine, polarisation or metabolic assays require explicit control of carrier conditions, serum or albumin binding, LPP activity, LPA species, exposure duration and replenishment strategy before nM and µM results can be compared directly.

Low-nanomolar or submicromolar LPA exposures have been used in some biological systems, but neither our group nor the available literature provides a receptor-resolved human primary macrophage dose–response study demonstrating TNF-alpha suppression by defined LPA species below 200 nM. Dose–response studies that directly test macrophage TNF-alpha, IL-10 and IL-6 responses across physiological concentrations of LPA, particularly 10–200 nM, are therefore not available in a form sufficient to validate the LPAR1-regulatory model in human disease. This unresolved point defines an important experimental priority for receptor-resolved human macrophage studies. Future work should test defined LPA species across 10 nM, 40 nM, 100 nM, 200 nM, 1 µM and 10 µM under controlled carrier conditions, using primary human monocyte-derived macrophages or disease-relevant tissue macrophages. Until such data are available, LPAR1-dependent macrophage regulation should not be generalized to human inflammatory disease.

A dose–response figure is therefore a priority for future experimental work rather than a claim that can be supported from the present literature. Representative experimental concentrations and translational caveats are summarised in Supplementary Table S1, shown as panels A and B. If suppression of inflammatory cytokines is observed only at 1–10 µM, the model should be restricted to sustained-exposure or high-local-exposure contexts whose physiological relevance depends on LPP activity, ATX-mediated replenishment, carrier conditions and exposure duration. If suppression is reproducible at 100–200 nM in primary human macrophages, the model would gain substantially greater translational plausibility.

Thus, future concentration-resolved macrophage experiments should not only test lower LPA concentrations, but also distinguish short-pulse receptor signalling from sustained exposure models and should report whether LPA is replenished or protected from degradation during the incubation.

## Immunometabolic interpretation

The following section is hypothesis-generating rather than a statement of a validated immunometabolic mechanism. Preliminary metabolic observations suggest that LPA may interact with macrophage metabolic programming, but the evidence is not yet sufficient to define a causal pathway. LPS-activated inflammatory macrophages commonly increase glycolysis, accumulate metabolic intermediates such as succinate and produce cytokines such as TNF-alpha, IL-1beta and IL-6. Regulatory or reparative macrophage states are often associated with mitochondrial oxidative phosphorylation, fatty-acid oxidation, efferocytosis and IL-10- or TGF-beta-associated programmes, although the classical M1/M2 framework is an oversimplification [66–72].

LPA could connect receptor signalling to immunometabolism through several plausible routes. LPAR1 can couple to Gi/o, Gq and G12/13 family pathways, allowing modulation of PI3K-Akt, MAPK, calcium flux, Rho/ROCK and cytoskeletal signalling [6–8]. In inflammatory macrophages, Gi-PI3K-Akt signalling could theoretically affect glucose uptake, mTOR activity, mitochondrial survival and cytokine feedback. AMPK-mTOR balance may provide another bridge between receptor signalling and oxidative metabolism. However, these connections remain inferential in the LPA macrophage context and should not be treated as established causal pathways.

In addition to macrophage models, LPA-associated metabolic reprogramming has been reported in murine CD8 T cells, where LPA signalling modulated T-cell metabolism and impaired antitumour immunosurveillance [17]. This observation supports the broader possibility that LPA can affect immune-cell metabolic state, but it should remain hypothesis-generating for macrophage biology because the cell type, receptor context and disease setting differ substantially.

The current author-group metabolic observations, including reduced lactate and increased ATP in RAW264.7 cells, are best described as preliminary metabolic readouts rather than proof of oxidative phosphorylation rescue [35]. They are not equivalent to Seahorse oxygen-consumption rate or extracellular-acidification rate data, isotope-tracing studies, mitochondrial-flux analysis or in vivo metabolic-flux measurements. This limitation usefully defines the next mechanistic step: LPA-related cytokine changes should be linked to direct metabolic-flux measurements before metabolic reprogramming is treated as causal. Therefore, this section does not claim that LPA definitively shifts macrophages from glycolysis to oxidative phosphorylation; rather, it identifies a mechanistic hypothesis that requires direct testing.

Future studies should measure oxygen consumption, extracellular acidification, glycolytic flux, fatty-acid oxidation, mitochondrial membrane potential, intracellular and extracellular ATP, adenosine, lactate, inflammasome activation and cytokine output in parallel. ATP signalling requires particular caution: increased intracellular ATP may indicate preserved mitochondrial metabolism, whereas extracellular ATP can activate P2X7 and amplify inflammasome-dependent inflammation [73]. Thus, the metabolic consequences of LPA may differ depending on whether ATP remains intracellular, is released extracellularly or is degraded to adenosine.

## Therapeutic challenges and clinical trial failures

The therapeutic targeting of the ATX–LPA axis is biologically attractive but clinically difficult. ATX inhibitors, LPA receptor antagonists and LPA analogues may theoretically modulate vascular leak, fibrosis, tumour immune escape or selected macrophage responses [14–17, 24, 32–35]. However, the same pathway can support physiological immune trafficking, tissue repair and vascular adaptation. To our knowledge, no interventional clinical trial has established LPA receptor antagonism as a therapeutic strategy for systemic autoimmune diseases such as SLE or rheumatoid arthritis.

A related translational problem is LPA receptor redundancy. Multiple LPA receptors can be expressed in the same tissue niche, and different receptors may produce overlapping, compensatory or even opposing outputs depending on cell type and activation state [6–8, 51]. Selective blockade of one receptor may therefore be insufficient if parallel receptors maintain LPA responses, whereas broad ATX inhibition or pan-pathway suppression may interfere with protective repair, vascular homeostasis or immune trafficking. This redundancy partly explains why receptor-selective therapeutic strategies require disease-phase-specific receptor mapping rather than simple extrapolation from one experimental receptor model.

The failure of ziritaxestat in idiopathic pulmonary fibrosis should be interpreted as a cautionary clinical signal rather than a simple problem of cell specificity [24]. More plausible explanations include redundancy of fibrotic pathways, advanced disease stage, insufficient or excessive target engagement, safety liabilities, compensatory lipid mediator networks, patient heterogeneity and the difficulty of modulating a pleiotropic lipid pathway in chronic fibrotic disease. The closest autoimmune-related example is systemic sclerosis, in which the LPA1 antagonist SAR100842 showed acceptable safety and evidence of target engagement but did not produce a statistically significant improvement in modified Rodnan skin thickness score [74].

In rheumatoid arthritis-associated interstitial lung disease, the LYSLUNG study addresses the ATX–LPA axis by measuring ATX and LPA in plasma and sputum, but it is an observational biomarker study rather than an LPA receptor antagonist trial [75]. Trials of LPA1 antagonists such as BMS-986020 in idiopathic pulmonary fibrosis are relevant to fibrotic biology but should not be used as proof-of-concept for autoimmune inflammation [76]. For autoimmune and myeloid-driven inflammatory diseases, the therapeutic bar is therefore high: the field must demonstrate regulatory effects in primary human macrophages at plausible concentrations, identify the responsible receptor subtype, define disease-phase specificity and exclude parallel activation of vascular leak or fibrotic programmes. A positive translational path remains possible, but it is likely to be narrower than broad ATX–LPA pathway inhibition. If the predictions proposed below are supported, therapeutic development should shift toward receptor-selective, disease-phase-specific and cell-context-aware modulation of LPA signalling, rather than systemic suppression or activation of the entire pathway.

## Related lysophospholipids and analogues

Related lysophospholipids provide useful comparators but should not be used as direct evidence for native LPA signalling. Cyclic phosphatidic acid, 2-carba cyclic phosphatidic acid, lysophosphatidylserine, lysophosphatidylinositol and lysophosphatidylcholine illustrate that lysophospholipid networks can influence inflammation, but their receptor usage, metabolic stability and downstream signalling differ from native LPA. Accordingly, this section is limited to contextual comparison; these analogues and related lipids should not be used as evidence for LPAR1-dependent macrophage regulation [52, 53, 77–82].

## Testable predictions and falsifiability

Prediction 1: Primary human macrophages with relatively dominant LPAR1 expression compared with LPAR5 will be more likely to show LPA-mediated attenuation of LPS-induced TNF-alpha than macrophages with LPAR5-dominant or balanced receptor expression. Because no validated numerical cutoff exists, this prediction should be tested using continuous receptor-expression ratios rather than an arbitrary threshold.

Prediction 2: If LPAR1-dependent macrophage regulation is physiologically relevant, LPA should attenuate LPS-induced TNF-alpha, IL-1beta or IL-6 in primary human macrophages at concentrations that include 100–200 nM. If suppression is observed only at 1–10 µM, the model should be restricted to sustained-exposure or high-local-exposure conditions whose physiological relevance depends on LPP activity, ATX-mediated replenishment, carrier conditions and exposure duration.

Prediction 3: Myeloid-cell-specific LPAR1 deletion should worsen chronic macrophage-driven inflammation if LPAR1 functions as an endogenous regulatory signal in that setting. If deletion has no effect, or improves disease, the LPAR1-regulatory model should be narrowed or rejected for that disease context.

Prediction 4: Acute injury models and chronic autoimmune models should show different LPA species profiles, receptor-expression patterns and myeloid outputs. If the same species and receptor profiles produce opposite effects without measurable differences in cell state or tissue context, the current framework would require revision.

Prediction 5: 18:1 LPA and 20:4 LPA may produce different cytokine and metabolic outputs at equimolar concentrations. If LPA species do not alter receptor engagement or macrophage responses, species-based explanations should be de-emphasised.

Prediction 6: Human disease cohorts should show coordinated relationships among ATX activity, LPA species, LPP expression, LPAR expression in immune subsets and cytokine profiles. If these layers do not correlate with disease phase or inflammatory phenotype, the clinical utility of the framework would be limited.

Prediction 7: If LPA species reflect disease phase in human neuroinflammation, CSF LPA profiles should differ between active and remitting inflammatory CNS disease and should not simply mirror nonspecific tissue injury. If CSF LPA species correlate only with injury or pain markers and not with inflammatory remission or myeloid-resolution markers, the clinical relevance of regulatory CNS LPA signalling should be narrowed.

Prediction 8: In rheumatoid arthritis, synovial-fluid ATX/LPA species and synovial-cell LPAR expression should stratify vascular–stromal remodelling from myeloid regulatory phenotypes. If LPA indices correlate with fibroblast-like synoviocyte migration, matrix remodelling or disease activity but not with macrophage IL-10 or resolution markers, LPA should be interpreted as a stromal-remodelling mediator rather than a myeloid-resolving signal in that disease.

These predictions make the framework vulnerable to experimental rejection. If they are not supported, the field should move away from broad claims of regulatory LPA signalling and instead restrict anti-inflammatory LPA biology to defined experimental conditions. This falsifiability is a strength of the framework: it converts current uncertainties into concrete priorities for receptor-resolved, concentration-resolved and human disease-oriented studies.

## Limitations of this review

This review has several limitations. First, it is a structured narrative review rather than a formal systematic review or meta-analysis, and therefore literature selection and evidence classification necessarily involved interpretive judgement. Second, the available literature remains uneven across LPA species, receptor subtype, concentration range, disease phase and human biospecimen context, which limits the strength of any general conclusion about anti-inflammatory or macrophage-regulatory LPA signalling. Third, receptor-resolved and concentration-resolved validation in primary human myeloid cells remains insufficient, particularly for defined LPA species tested under controlled carrier and degradation conditions. Finally, this review includes experimental studies from the authors’ programme; these studies are explicitly identified as author-group limited where relevant and are treated as hypothesis-generating rather than definitive translational evidence.

## Future directions

Future work should prioritise receptor-resolved and concentration-resolved studies in primary human myeloid cells. Experimental designs should compare individual LPA species, carrier conditions, receptor antagonism or genetic perturbation and macrophage activation states. Human studies should combine lipidomics, ATX and LPP measurements, immune-cell receptor profiling, cytokine data and disease-phase annotation.

The aim is not to promote LPA as a broad explanatory hub for inflammation, but to define the conditions under which LPA signalling becomes functionally relevant, clinically actionable and experimentally testable.

In organ-specific disease, the key question is whether local LPA biology aligns with vascular–stromal injury programmes or with regulatory myeloid responses. For lupus nephritis and rheumatoid arthritis, future translational studies should combine species-resolved LPA lipidomics with LPAR1–LPAR6 expression profiling in macrophage and stromal compartments, ideally using spatial transcriptomics, single-cell RNA sequencing and disease-activity measures. At present, these studies should be described as critical translational priorities rather than established or ongoing validation.

Spatial lipidomics provides a complementary route to this goal. MALDI mass spectrometry imaging and related approaches can map regional lipid distributions in tissue sections, and on-tissue derivatization strategies have improved the detection of phosphate-containing bioactive lipids such as LPA and sphingosine-1-phosphate [83, 84]. Integrating spatial lipidomics with spatial transcriptomics could identify whether defined LPA species accumulate within macrophage-rich inflammatory niches and whether these lipid microenvironments align with LPAR expression, macrophage state, stromal remodelling or vascular injury. However, this approach remains technically challenging because individual LPA species are low-abundance, susceptible to post-sampling metabolism, prone to ion-suppression and isobaric or isomeric ambiguity, and difficult to quantify absolutely in situ. Robust validation will therefore require careful tissue handling, internal standards, MS/MS confirmation, co-registration with adjacent spatial transcriptomic or histological sections, and parallel bulk LC–MS/MS measurements.

Non-canonical GPCR scaffolding pathways may be worth testing, but direct evidence for their involvement in LPA-driven macrophage regulation is currently lacking. These modules should therefore remain outside the main mechanistic model until receptor-specific interaction, loss-of-function and rescue experiments are available.

## Conclusion

LPA is best understood as a context-dependent inflammatory mediator rather than as a uniformly pathogenic or anti-inflammatory lipid. The strongest evidence supports LPA as a driver of leukocyte recruitment, vascular leak, fibrosis, pain-associated neural injury, pathogenic immune suppression in cancer and tissue remodelling. Independent studies also indicate that LPA can attenuate selected LPS/TLR4-driven macrophage responses, including IL-10 induction and TNF-alpha suppression. However, these anti-inflammatory effects are receptor-divergent and incompletely translated, and the more specific proposal that LPAR1 drives M2-like and metabolic macrophage reprogramming remains author-group limited.

Human biofluid data currently associate LPA more strongly with injury, pain and tissue remodelling than with inflammation resolution. No human disease study has yet demonstrated that LPA drives resolution-phase myeloid regulation or M2 macrophage polarisation in vivo. These limitations should be treated as constraints on, rather than validation of, anti-inflammatory LPA models, and they define the most important next step for translation. Anti-inflammatory LPA models should be tested directly in primary human myeloid cells, at physiologically plausible concentrations, using defined LPA species and receptor-resolved perturbation. The field should now move from asking whether LPA is pro- or anti-inflammatory to identifying the variables that determine LPA output: receptor subtype, cell type, disease phase, lipid species, local concentration, metabolic state and tissue niche.

## Supplementary Information

Below is the link to the electronic supplementary material.

Supplementary file1 (DOCX 16 KB)

## Data Availability

No datasets were generated or analysed during the current study.

## Abbreviations

AMPK: AMP-activated protein kinase
ATX: Autotaxin
cPA: Cyclic phosphatidic acid
CSF: Cerebrospinal fluid
ECAR: Extracellular acidification rate
ERK: Extracellular signal-regulated kinase
FLS: Fibroblast-like synoviocyte
GPCR: G protein-coupled receptor
IBD: Inflammatory bowel disease
IL: Interleukin
IPF: Idiopathic pulmonary fibrosis
LPA: Lysophosphatidic acid
LPAR: Lysophosphatidic acid receptor
LPC: Lysophosphatidylcholine
LPP: Lipid phosphate phosphatase
LPS: Lipopolysaccharide
MAPK: Mitogen-activated protein kinase
MS: Multiple sclerosis
mTOR: Mechanistic target of rapamycin
OCR: Oxygen-consumption rate
PI3K: Phosphoinositide 3-kinase
RA: Rheumatoid arthritis
ROCK: Rho-associated protein kinase
SF: Synovial fluid
SLE: Systemic lupus erythematosus
TBI: Traumatic brain injury
TGF-beta: Transforming growth factor-beta
TLR4: Toll-like receptor 4
TNF-alpha: Tumour necrosis factor-alpha
