# Supplementary material for: Context-dependent lysophosphatidic acid signalling in inflammation: evidence hierarchy, myeloid regulation and translational gaps
Source: Inflamm Res. 2026 Aug 4;75(1):187. doi: 10.1007/s00011-026-02337-z (PMC13437610; doi:10.1007/s00011-026-02337-z)
Supplement: Supplementary file 1 [file 11_2026_2337_MOESM1_ESM.docx]

# Supplementary material

**Supplementary Table S1. Representative LPA concentrations and translational caveats, shown in two panels**

**Supplementary Table S1A. Experimental macrophage studies and CNS injury comparator**

| **Study/context** | **Cell/sample** | **LPA species and concentration** | **Main readout** | **Human comparator and caveat** |
| --- | --- | --- | --- | --- |
| Independent LPS/TLR4 macrophage-regulation studies | Primary macrophages, J774 cells, peritoneal macrophages | Experimental LPA; often pharmacological; receptor-divergent | IL-10 up-regulation, TNF-alpha suppression, reduced endotoxin responses | Supports broad anti-inflammatory phenomenon; not direct LPAR1 replication; concentration gap remains |
| Author-group LPAR1 macrophage study | RAW264.7 macrophages | Reported pharmacological LPA exposure | Cytokine suppression, M2-like markers, metabolic readouts | Author-group limited; needs independent primary-cell and low-dose validation |
| Human CSF traumatic brain injury | Patient CSF and experimental TBI | CSF LPA elevation after injury | Biomarker/injury association; antibody intervention in model | Supports injury-associated pathogenic LPA signalling rather than anti-inflammatory translation |

**Supplementary Table S1B. Human biofluid contexts and future validation experiment**

| **Study/context** | **Cell/sample** | **LPA species and concentration** | **Main readout** | **Human comparator and caveat** |
| --- | --- | --- | --- | --- |
| Lumbar spinal stenosis/neuropathic pain context | Patient CSF | Increased LPA-related species | Pain or symptom association | Constrains broad neuroprotective interpretations |
| Rheumatoid arthritis synovial fluid | Human synovial fluid/synovial tissue | LPA/ATX-associated biology | Fibroblast-like synoviocyte migration and tissue remodelling | Supports stromal activation rather than myeloid resolution |
| Proposed future experiment | Primary human macrophages | 10 nM, 40 nM, 100 nM, 200 nM, 1 µM, 10 µM; defined species | Receptor-resolved cytokine and metabolic readouts | Required to test physiological plausibility |
